# Darcy-Forchheimer hybrid nanofluid flow over a stretching curved surface with heat and mass transfer

**DOI:** 10.1371/journal.pone.0249434

**Published:** 2021-05-07

**Authors:** Anwar Saeed, Wajdi Alghamdi, Safyan Mukhtar, Syed Imad Ali Shah, Poom Kumam, Taza Gul, Saleem Nasir, Wiyada Kumam

**Affiliations:** 1 Faculty of Science, Center of Excellence in Theoretical and Computational Science (TaCS-CoE), King Mongkut’s University of Technology Thonburi (KMUTT), Thung Khru, Bangkok, Thailand; 2 Faculty of Computing and Information Technology, Department of Information Technology, King Abdulaziz University, Jeddah, Saudi Arabia; 3 Department of Basic Sciences, Deanship of Preparatory Year, King Faisal University, Hofuf, Al Ahsa, Saudi Arabia; 4 Mathematics Department, City University of Science and Information Technology, Peshawar, Pakistan; 5 Department of Medical Research, China Medical University Hospital, China Medical University, Taichung, Taiwan; 6 Department of Mathematics, Abdul Wali Khan University, Mardan, Khyber, Pakhtunkhwa, Pakistan; 7 Faculty of Science and Technology, Department of Mathematics and Computer Science, Program in Applied Statistics, Rajamangala University of Technology Thanyaburi, Thanyaburi, Pathumthani, Thailand; Central University of Karnataka, INDIA

## Abstract

The present article provides a detailed analysis of the Darcy Forchheimer flow of hybrid nanoliquid past an exponentially extending curved surface. In the porous space, the viscous fluid is expressed by Darcy-Forchheimer. The cylindrical shaped carbon nanotubes (SWCNTs and MWCNTs) and Fe_3_O_4_ (iron oxide) are used to synthesize hybrid nanofluid. At first, the appropriate similarity transformation is used to convert the modeled nonlinear coupled partial differential equations into nonlinear coupled ordinary differential equations. Then the resulting highly nonlinear coupled ordinary differential equations are analytically solved by the utilization of the “Homotopy analysis method” (HAM) method. The influence of sundry flow factors on velocity, temperature, and concentration profile are sketched and briefly discussed. The enhancement in both volume fraction parameter and curvature parameter *k* results in raises of the velocity profile. The uses of both Fe_3_O_4_ and CNTs nanoparticles are expressively improving the thermophysical properties of the base fluid. Apart from this, the numerical values of some physical quantities such as skin friction coefficients, local Nusselt number, and Sherwood number for the variation of the values of pertinent parameters are displayed in tabular forms. The obtained results show that the hybrid nanofluid enhances the heat transfer rate 2.21%, 2.1%, and 2.3% using the MWCNTs, SWCNTs, and Fe_3_O_4_ nanomaterials.

## 1. Introduction

The flow analysis over a stretch sheet is essential for use in many engineering and industrial sectors. Its fascinating applications are utilized in the production of plastic and rubber sheets, metalworking processes such as hot rolling, aerodynamic extrusion of plastic sheets, melt spinning as a metal forming technique, elastic polymer substance, and emollient production, paints, production of glass-fiber, etc. The first analysis on the flow overextending plane surface was made by Crane [1]. Generally, Crane’s suggested model of the linearly stretching plate is not used in many industrial sectors. So, researchers find an interest in investigating the various aspects of the stretching rate. Later on, following Crane’s idea, many researchers investigate several characteristics of this type of flow over the stretching sheet [2]. Sajid et al. [3] contemplated micropolar fluid and boundary layer flow and concluded that the dimensionless curvature results in the improvement of the layer size. Imtiaz et al. [4] illustrated MHD flow with heterogeneous-homogeneous reactions due to the stretching curved surface. It is evident that the temperature and velocity of fluid rise for higher curvature. Rosca et al. [5] examined fluid flow due to contracting and extending sheets. Hayat et al. [6] numerically investigated the Darcy-Forchhemier flow of extending bent surface with Cattaneo-Christov theory. Kumar et al. [7] deliberated the radiation effect on MHD fluid (Casson) flow over the exponentially curved sheet. Hayat et al. [8] and Maria et al. [9] investigate ferro liquid flow with homogeneous-heterogeneous effect over the curled widening surface. The viscous fluid flow with the power-law over the extending bent surface was examined by Sanni et al. [10]. The nano-silver and diamond thermos physical characteristics simulations over curved surface flow were investigated by Khan et al. [11]. Some other relevant and innovative investigations under different conditions are discussed in [12–15].

Nanofluids have attracted significant interest from researchers due to their motivating heat transfer in numerous manufacturing and engineering applications. Traditional operating fluids such as water, engine oils, and ethylene glycol have reduced thermal efficiency that restricts their use in modern cooling applications. Nanofluids comprise nanoparticles such as copper, alumina, carbides, nitrides, metal oxides, graphite, and carbon nanotubes that improve the thermal conductivity of base fluids. These nanofluids are commonly used in modern heating and cooling systems, solar panels, the latest fuel generation, hybrid-powered vehicles, cancer treatment, drug delivery, and medicine. A variety of experiments related to the flow of nanofluids have been performed due to these various applications of nanofluids. Thermal conductivity and convective heat transfer are enhanced by the suspension of nanoparticles in the base fluid. Choi [16] initially adopted this concept and introduced a revolutionary new form of nanofluid that represents high thermal conductivity.

The heat transfer of nano-carbon fluid has received considerable interest in the last two decades due to its extensive uses in the fields of nanotechnology and medicine. CNTs are the simple chemical structure along with the composition of carbon atoms, rolled in cylindrical form. CNTs have extraordinary thermophysical, chemical, electrical, and mechanical features that can be utilized efficiently as a nanoparticle in the base fluid. They have unique advantages because of tiny tube-sized structures, such as large surface area, configuration, chemical stability, hardness, and their smallest dimension over other nanoparticles. CNTs depend on the number of graphene layers, which subdivided it into (SWCNTs) single-walled and (MWCNTs) multi-walled carbon nanotubes, respectively. Haq et al. [17] examined the numerical outcomes for conducting fluid and heat exchange due to carbon nanotubes merged in various based fluids over an extending sheet using a numerical scheme. The influence of carbon nanotubes water-based nano liquid (micropolar) on heat transport and magneto-hydrodynamics (MHD) flow between two rotating discs using the HAM technique was employed by Rahman et al. [18]. Mathanthash et al. [19] studied the heat source’s impact and exponential space during the nanofluid Flow. The entropy generation between two rotating disks of CNTs nanofluid flow in the occurrence of thermal radiation and MHD was inspected by Hosseinzedeh et al. [20]. Ding et al. [21] explored the heat transfer using (MWCNTs) in the base liquid. Akber et al. [22] described the magnetic field’s influence over CNTs nanofluid flow through a moving permeable channel.

Hybrid nanofluids have become a new class of working fluids that comprise extremely small particles less than 100 nm in size and are important in heat transfer applications. These fluids consist of two solid materials, such as Al_2_O_3_–Cu, SWCNTs-Fe_3_O_4,_ MWCNTs-Fe_3_O_4_, Al_2_O_3_–Ag, Cu–TiO_2_ and Cu–CuO, in traditional liquids (water, kerosene, ethylene glycol, and engine oil). The purpose of the use of hybrid nanofluids is to further improve the flow of heat and grow nanotechnology. Nowadays, these hybrid nanofluids are implemented in numerous heat transfer applications as mini channel heat sinks, air conditioning systems, micro channels, helical coil heat exchangers, tubular and plate heat exchangers. An extensive research on hybrid nanofluids can be found in the literature for example Gabli et al. [23] have examined the *Fe*_3_*O*_4_ nanoparticles dispersion in the non-Newtonian fluids for heat transfer enhancement. The status of hybrid nanofluid in the heat development rate has been premeditated by Nadeem et al. [24]. Chamkha et al. [25] scrutinized the transmission of heat and magnetohydrodynamic Flow of hybrid nanofluids utilizing a rotating frame. Sundar et al. [26] explored the heat transfer features of Graphene Oxide /*CO*_3_*O*_4_ hybrid nanofluids. Wei et al. [27] estimated the thermophysical properties of oil-based hybrid nanofluids for heat transferal uses. Momin [28] investigated analytically the various aspects of diverse convective laminar hybrid nanoliquid flow in a tending cylinder. Sundar et al. [29] inspected the greater temperature energy transfers and the friction feature of the hybrid nano liquid. Some latest studies on different flow geometries of hybrid nanofluid are listed in Refs. [30–33].

The novelty of the model is pointed out as:

- The heat and mass transfer at the same time for the solid nanoparticles initiated, while in the existing literature, only the heat transfer is considered.
- The (*CNT*_*S*_+*Fe*_3_*O*_4_/*H*_2_*O*) hybrid nanofluid flow due to an extending surface for the heat and mass transfer is considered while no one has considered the (*CNT*_*S*_+*Fe*_3_*O*_4_/*H*_2_*O*) hybrid nanofluid for the same model.
- Our motivation for the current work, to investigate and model the Darcy-Forchheimer water-based (*CNT*_*S*_+*Fe*_3_*O*_4_) hybrid nano liquid flow due to an extending curved surface.
- The priority to initiate the Xue [34] and Saba et al. [35] theoretical models for such type of Flow and set up an arrangement for velocity and the temperature profile opted “Homotopy analysis method” (HAM).
- We have protracted the idea of Hayat et al. [36] by including the above-mentioned features.

## 2. Problem formulation

**Fig 1:**
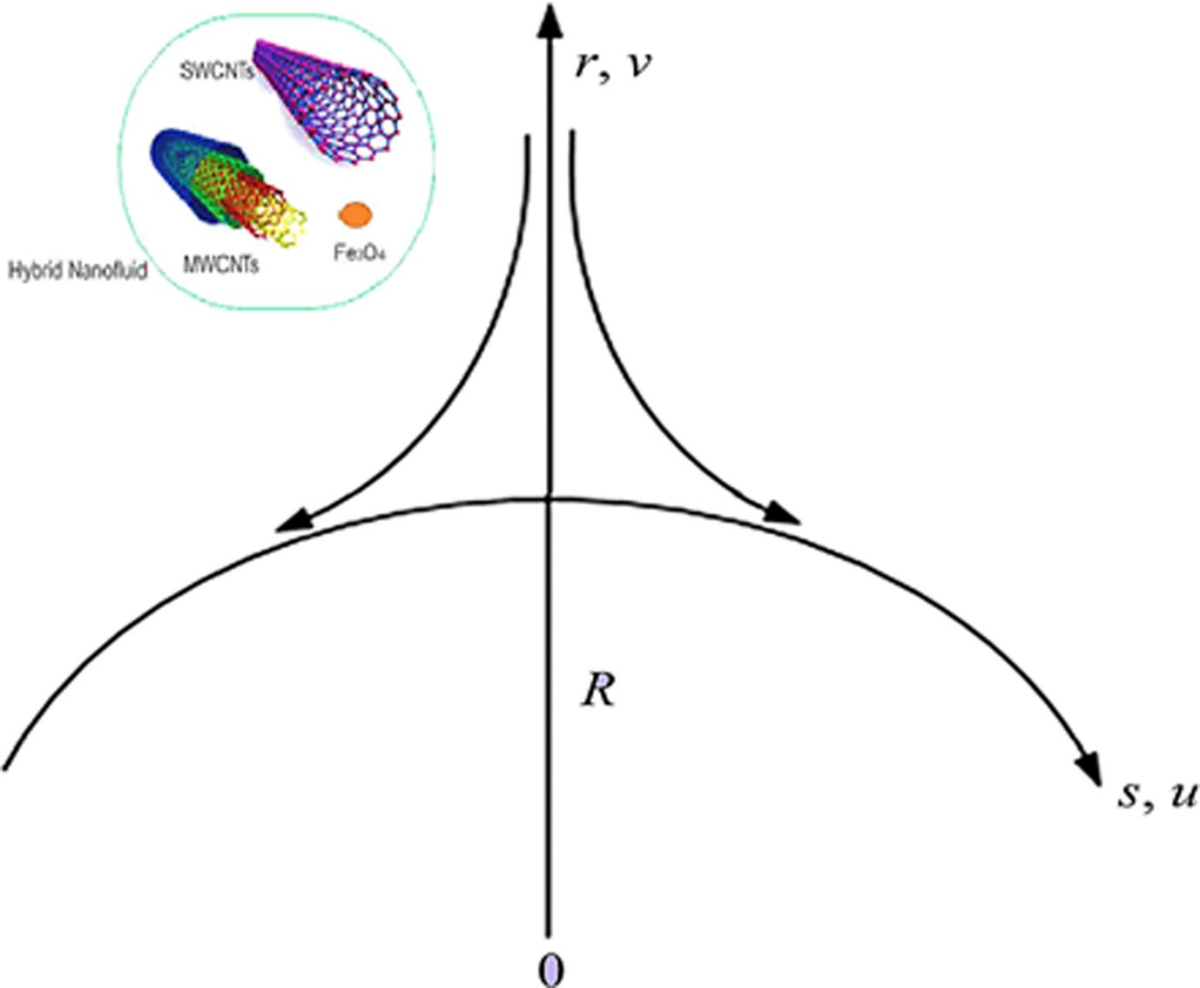


Let us consider the steady and two-dimensional Darcy Forchheimer flow of hybrid nano liquid due to an extending curved surface. In permeable space, the viscous fluid is expressed by Darcy-Forchheimer. The flow is induced through the exponential folded sheet, spiral in a circle with radius *R*, illustrated in Fig 1. Here (*u*, *v*) is taken as the velocity component and (*r*, *s*) is the space coordinate. The expression *U*_*w*_(*s*) = *ae*^*s*/*L*^ depicts the exponential stretching velocity, while *T*_∞_ is ambient and *T*_*w*_ is the curved surface temperature. Keep in view, the above description of the velocity and energy equation along with their boundary condition is formulated as [37,38]:

$$\frac{\partial }{\partial r}\left(\left(r+u\right)v\right)+R\frac{\partial u}{\partial r}=0,$$ (1)

$$\frac{u^{2}}{r+R}=\frac{1}{\rho_{hnf}}\frac{\partial p}{\partial r},$$ (2)

$$\begin{array}{l} v\frac{\partial u}{\partial r}+\frac{R}{r+R}u\frac{\partial u}{\partial s}+\frac{uv}{r+R}=-\frac{1}{\rho_{hnf}}\frac{R}{r+R}\frac{\partial p}{\partial s}+\\ v_{hnf}\left(\frac{\partial^{2}u}{\partial r^{2}}+\frac{1}{r+R}\frac{\partial u}{\partial r}-\frac{u}{{\left(r+R\right)}^{2}}\right)-\frac{v_{hnf}}{K^{*}}u-Fu^{2}, \end{array}$$ (3)

$$\left[v\frac{\partial T}{\partial r}+u\frac{\partial T}{\partial s}\frac{R}{r+R}\right]=\alpha_{hnf}\left(\frac{\partial^{2}T}{\partial r^{2}}+\frac{1}{r+R}\frac{\partial T}{\partial r}\right),$$ (4)

$$\left[v\frac{\partial C}{\partial r}+u\frac{\partial C}{\partial s}\frac{R}{r+R}\right]=D_{hnf}\left(\frac{\partial^{2}C}{\partial r^{2}}+\frac{1}{r+R}\frac{\partial C}{\partial r}\right)$$ (5)

Where *u*, *v* represent the component of velocity in *s* and *r*-direction. Here, $F=\frac{C_{b}}{sK^{*1/2}}$, is the non-uniform inertia coefficient and K* is the porous space permeability.

The basic flow conditions are:

$$\begin{array}{l} v=0,u=U_{w}\left(s\right)={\text{ae}}^{s/L},\text{T}={\text{T}}_{\infty }+T_{0}e^{As/2L}={\text{T}}_{w},C=C_{\infty }+C_{0}e^{As/2L}=C_{w},\;\text{at}\;r=0,\\ u\to 0,\frac{\partial u}{\partial r},\text{T}\to T_{\infty },C\to C_{\infty },\;\text{at}\;r\to \infty \end{array}$$ (6)

### 2.1 Expressions for hybrid nanofluid

**Table 1:** The Thermophysical properties of CNTs, Fe_3_O_4_ and base fluid water [19,29].

Thermophysical properties	*ρ*(kg/m^3^)	*C*_*p*_(j/kgK)	*k*(W/mK)
**SWCNTs**	2600	425	6600
**MWCNTs**	1600	796	300
**Fe**_**3**_**O**_**4**_	5200	670	6
**Pure water**	997.1	4179	0.613

$$\begin{array}{l} \upsilon_{hnf}=\frac{\mu_{hnf}}{\rho_{hnf}},\mu_{hnf}=\frac{\mu_{f}}{{\left(1-\phi_{1}\right)}^{5/2}{\left(1-\phi_{2}\right)}^{5/2}},\frac{{\left(\rho \right)}_{hnf}}{{\left(\rho \right)}_{f}}=\left(1-\phi_{2}\right)\left\{1-\left(1-\frac{\left(\rho \right)Ms}{{\left(\rho \right)}_{f}}\right)\phi_{1}\right\}+\frac{{\left(\rho \right)}_{CNT}}{{\left(\rho \right)}_{f}}\phi_{2},\\ \frac{{\left(\rho {\text{C}}_{p}\right)}_{hnf}}{{\left(\rho {\text{C}}_{p}\right)}_{f}}=\left(1-\phi_{2}\right)\left\{1-\left(1-\frac{\left(\rho {\text{C}}_{p}\right)Ms}{{\left(\rho {\text{C}}_{p}\right)}_{f}}\right)\phi_{1}\right\}+\frac{{\left(\rho {\text{C}}_{p}\right)}_{CNT}}{{\left(\rho {\text{C}}_{p}\right)}_{f}}\phi_{2},\\ \frac{k_{hnf}}{k_{bf}}=\frac{1-\phi_{2}+2\phi_{2}\frac{k_{CNT}}{\left({\text{k}}_{CNT}-{\text{k}}_{bf}\right)}-\ln\frac{k_{CNT}+k_{bf}}{2k_{bf}}}{1-\phi_{2}+2\phi_{2}\frac{k_{bf}}{\left({\text{k}}_{CNT}-{\text{k}}_{bf}\right)}-\ln\frac{k_{CNT}+k_{bf}}{2k_{bf}}},\frac{k_{bf}}{k_{f}}=\frac{k_{MS}+{\left(m-1\right)}_{kf}-\left(m-1\right)\phi_{1}\left({\text{k}}_{f}-{\text{k}}_{MS}\right)}{k_{MS}+{\left(m-1\right)}_{kf}-\phi_{1}\left({\text{k}}_{f}-{\text{k}}_{MS}\right)}, \end{array}$$ (7)

Pr and Sc indicated the Prandtl and Schmidt number. (C_*p*_)_*MS*_,*ρ*_*MS*_ and *ρ*_*CNT*_ specified capacitance and densities of *Fe*_3_*O*_4_ and CNTs respectively. The *k*_*hnf*_, *k*_*f*_ is the thermal conductivity of nanofluids *Fe*_3_*O*_4_ and carrier fluid *H*_2_*O*.The density of base fluid *H*_2_*O*, specific heat and viscosity are illustrated via *ρ*_*f*_, (*ρ*_*p*_)_*f*_ and *μ* respectively. The volumetric concentration of *Fe*_3_*O*_4_ and carbon nanotubes are denoted by *ϕ*_1_ and *ϕ*_2_.

The appropriate transformation is [37–39]:

$$\begin{array}{l} \eta ={\left(\frac{ae^{s/L}}{2\upsilon_{f}L}\right)}^{1/2}r,v=-\frac{R}{r+R}\sqrt{\frac{av_{f}e^{s/L}}{2L}}\left(f\left(\eta \right)+\eta f\prime \left(\eta \right)\right),\text{u}=U_{w}=ae^{s/L}f\prime \left(\eta \right),\\ \text{p}=\rho_{f}{\text{a}}^{2}{\text{e}}^{2s/L}\text{H}\left(\eta \right),T=T_{\infty }+T_{0}e\frac{As}{2L}\Theta \left(\eta \right),C=C_{\infty }+C_{0}e\frac{As}{2L}\Phi \left(\eta \right). \end{array}$$ (8)

Utilizing the above similarity transformations, the present governing equations turns into nonlinear coupled ordinary differential equations, which are presented as follows

$$H\prime =\left(\frac{{\left(\rho \right)}_{hnf}}{{\left(\rho \right)}_{f}}\right)\frac{1}{\eta +K}f\prime^{2},$$ (9)

$$\begin{array}{l} \left(f^{\prime \prime \prime }+\frac{1}{\eta +K}f^{\prime \prime }-\frac{1}{{\left(\eta +K\right)}^{2}}f\prime -2\lambda f\prime \right)-{\left(1-\phi_{1}\right)}^{2.5}{\left(1-\phi_{2}\right)}^{2.5}\left(\frac{{\left(\rho \right)}_{hnf}}{{\left(\rho \right)}_{f}}\right)\\ \left(\frac{\eta +2K}{{\left(\eta +K\right)}^{2}}K{\left(f\prime \right)}^{2}-\frac{K}{\eta +K}ff^{\prime \prime }-\frac{K}{{\left(\eta +K\right)}^{2}}+2Frf\prime^{2}\right)={\left(1-\phi_{1}\right)}^{2.5}{\left(1-\phi_{2}\right)}^{2.5}\frac{K}{\eta +K}\left(4H+\eta H\right). \end{array}$$ (10)

$$\frac{k_{hnf}}{k_{f}}\left(\Theta^{\prime \prime }+\frac{1}{\eta +K}\Theta \prime \right)+\left(\frac{{\left(\rho {\text{C}}_{p}\right)}_{hnf}}{{\left(\rho {\text{C}}_{p}\right)}_{f}}\right)\mathrm{Pr}\frac{K}{\eta +K}\left(f\Theta \prime -Af\prime \Theta \right)=0,$$ (11)

$$\left(1-\phi_{1}\right)\left(1-\phi_{2}\right)\left(\Phi^{\prime \prime }+\frac{1}{\eta +K}\Phi \prime \right)+Sc\left(\frac{K}{\eta +K}f\Phi \prime \right)=0.$$ (12)

By eliminating *H* from Eqs (9) and (10), we get

$$\begin{array}{l} \left[f\prime^{v}+\frac{2}{\eta +K}f^{\prime \prime \prime }-\frac{1}{{\left(\eta +K\right)}^{2}}f^{\prime \prime }+\frac{1}{{\left(\eta +K\right)}^{3}}f\prime -2\lambda \left(f^{\prime \prime }+\frac{1}{\left(\eta +K\right)}f\prime \right)\right]+\\ \frac{{\left(\rho \right)}_{hnf}}{{\left(\rho \right)}_{f}}\left[\begin{array}{l} \frac{K}{{\left(\eta +K\right)}^{2}}ff^{\prime \prime }+\frac{K}{\left(\eta +K\right)}ff^{\prime \prime \prime }-\frac{K}{{\left(\eta +K\right)}^{3}}ff^{\prime }\frac{3K}{{\left(\eta +K\right)}^{2}f\prime^{2}}-\frac{3K}{\left(\eta +K\right)}f^{\prime }f^{\prime \prime }\\ -2Fr\left(2f^{\prime }f^{\prime \prime }+\frac{1}{\eta +K}f^{\prime }{}^{2}\right) \end{array}\right]=0, \end{array}$$ (13)

The transform conditions for nonlinear differential equations are:

$$\begin{array}{l} f=0,f\prime =1,\Theta =1,\Phi =1,\text{at}\;\eta =0.\\ f\prime \to 0,f^{\prime \prime }\to 0,\Theta \to 0,\Phi \to 0,\;\text{at}\;\eta =\infty . \end{array}$$ (14)

In the above transform equation *Fr* depicts Forchheimer number, *Pr* is the Prandtl number, *K* is the Curvature parameter, *λ* is the local porosity parameter and Schmidt number are defined as:

$$Fr=\frac{C_{b}}{K^{*1/2}},\mathrm{Pr}=\frac{\upsilon_{f}}{\alpha_{f}},K=\left(\frac{ae^{s/L}}{2\upsilon_{f}L}\right),\lambda =\frac{\upsilon_{f}L}{K^{*}U_{w}},Sc=\frac{\upsilon_{f}}{D_{f}}.$$ (15)

### 2.2 Quantities of physical interest

The expression for the local Nusselt number, Sherwood Number and Skin friction are given as

LS(Re2)−12Nux=−khnfkbfΘ′(0),LS(Re2)−12Shx=−Φ′(0),Re2Cfx=1(1−ϕ1)2.5(1−ϕ2)2.5f′′(0). (16)

Where local Reynolds number is

Rex=u0x2vl. (17)

## 3. Problem solution

The problem is solved through the HAM technique, which was offered by Liao [40–42]. The initial approximation for velocity *f*_0_ and temperature Θ_0_ are given a

$$f_{0}\left(\eta \right)=e^{-\eta }-e^{-2\eta },\Theta_{0}\left(\eta \right)=e^{-\eta },\;\Phi_{0}\left(\eta \right)=e^{-\eta }.$$ (18)

The linear operatives are offered as:

$$L_{f}\left(\text{f}\right)=f\prime^{v}\;\text{and}\;L_{\Theta }\left(\Theta \right)=\Theta^{\prime \prime }.$$ (19)

The expand form of $L_{f}$, $L_{\Theta }$ and $L_{\Phi }$ are:

$$L_{f}\left[\chi_{1}+\chi_{2}\eta +\chi_{3}\eta^{2}+\chi_{4}\eta^{3}\right]=0,\;L_{\Theta }\left[\chi_{5}+\chi_{6}\eta \right]=0,\;L_{\Phi }\left[\chi_{7}+\chi_{8}\eta \right]=0.$$ (20)

### 3.1 OHAM convergence

The convergence of the OHAM method has been obtained using the idea of Liao [40,42].

$$\varepsilon_{m}^{f}=\frac{1}{l+1}{\sum_{j=1}^{l}\left[N_{f}{\left(\sum_{k=1}^{m}f\left(\eta \right)\right)}_{\eta =j\delta \eta }\right]}^{2},$$ (21)

$$\varepsilon_{m}^{\Theta }=\frac{1}{l+1}{\sum_{j=1}^{l}\left[N_{\Theta }{\left(\sum_{k=1}^{m}f\left(\eta \right),\sum_{k=1}^{,m}\Theta \left(\eta \right)\right)}_{\eta =j\delta \eta }\right]}^{2},$$ (22)

$$\varepsilon_{m}^{\Phi }=\frac{1}{l+1}{\sum_{j=1}^{l}\left[N_{\Phi }{\left(\sum_{k=1}^{m}\Phi \left(\eta \right)\right)}_{\eta =j\delta \eta }\right]}^{2},$$ (23)

The whole sum of the square residual is clear as $\varepsilon_{m}^{t}=\varepsilon_{m}^{f}+\varepsilon_{m}^{\Theta }+\varepsilon_{m}^{\Phi }$.

**Table 2:** The total residual errors.

*m*	$\varepsilon_{m}^{t}SWCNTs$	$\varepsilon_{m}^{t}SWCNTs$	$\varepsilon_{m}^{t}Fe_{3}O_{4}$
**5**	1.8168 × 10^−4^	1.9479 × 10^−4^	1.4257 × 10^−4^
**13**	1.1223 × 10^−5^	1.2354 × 10^−5^	1.18312 × 10^−5^
**23**	1.3599 × 10^−6^	0.4698 × 10^−6^	0.4489 × 10^−6^
**30**	3.2578 × 10^−7^	4.3689 × 10^−7^	4.1464 × 10^−7^

When *Fr* = *k* = 0.6, *ϕ*_1_ = 0.02, *ϕ*_2_ = 0.2, *λ* = 0.2, Pr = 6.3, A = 0.4.

**Table 3:** Present study and Hayat et al. [36].

	Hayat et al. [35]	Present
*f*”(0)	0.735	0.7352130
−Θ’(0)	-1.375	-1.3752410
−Φ’(0)	………….	-1.3620189

**When** *ϕ*_1_ = *ϕ*_2_ = *ϕ* = 0, *Fr* = *k* = 0.6, *λ* = 0.2, Pr = 6.3, A = 0.4.

**Table 4:** The heat transfer has been calculated percent wise as for the various nanoparticles Pr = 6.2, *λ* = 0.02, *A* = 0.2, *Fr* = 0.3, using the % formula $\%\;\text{Increase}=\frac{\text{With}\;\text{Nanoparticle}}{\text{Without}\;\text{Nanoparticle}}\times 100={\text{Result}}_{,}\text{Result}-100\%=\%\text{enhancment}$.

*ϕ*_1_ = *ϕ*_2_	Θ’(0) *MWCNTs*	Θ’(0) *SWCNTs*	Θ’(0) *Fe*_3_*O*_4_
0.0	1.1593	1.1482	1.1535765
0.01	1.1737999 (1.2507% Increase)	1.1636898 (1.3490% Increase)	1.559517 (2.11% Increase)
0.02	1.1849894 (2.215% Increase)	1.1732025 (2.1775% Increase)	1.1898694 (3.1461% Increase)

## 4. Results and discussion

**Fig 2:**
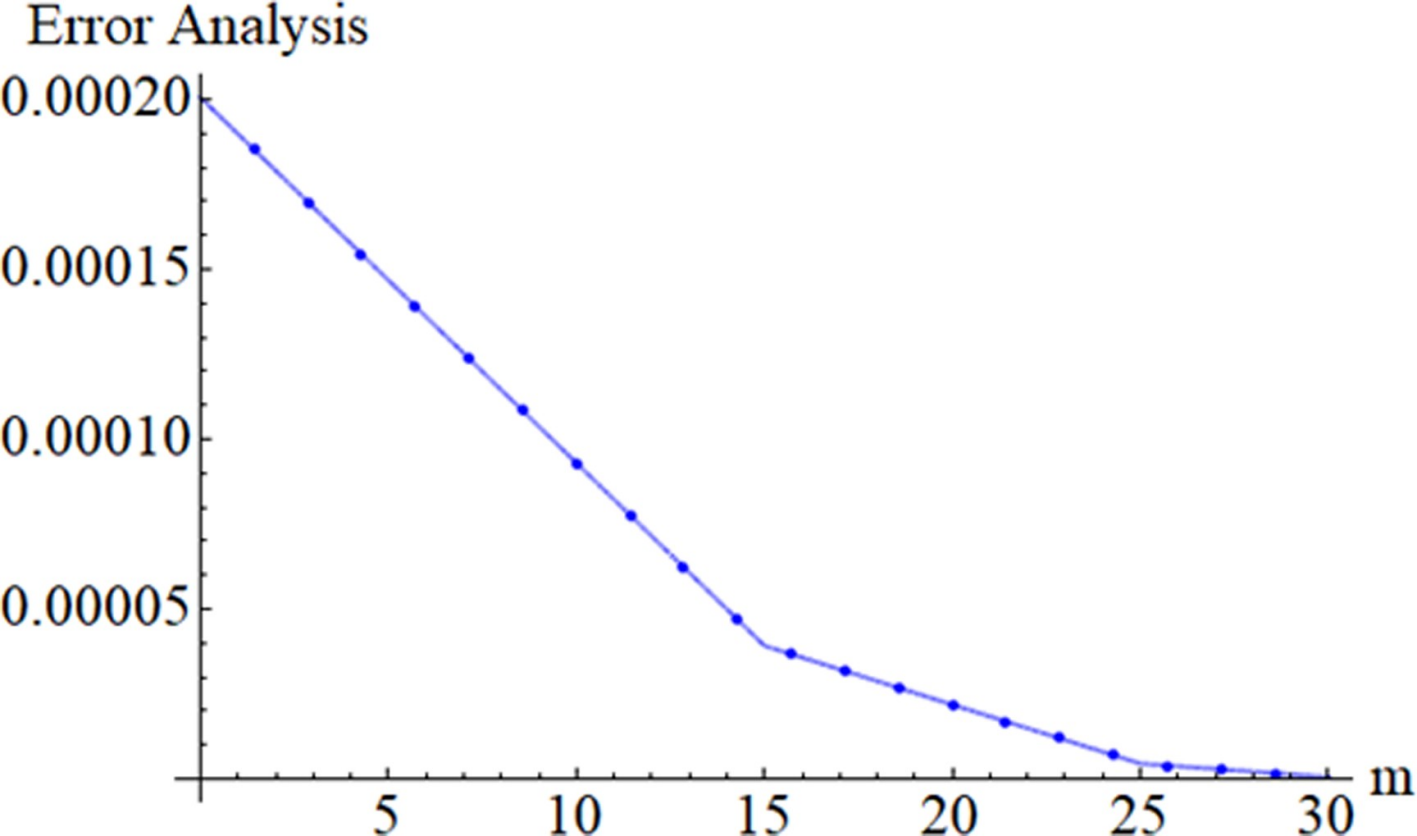


**Fig 3:**
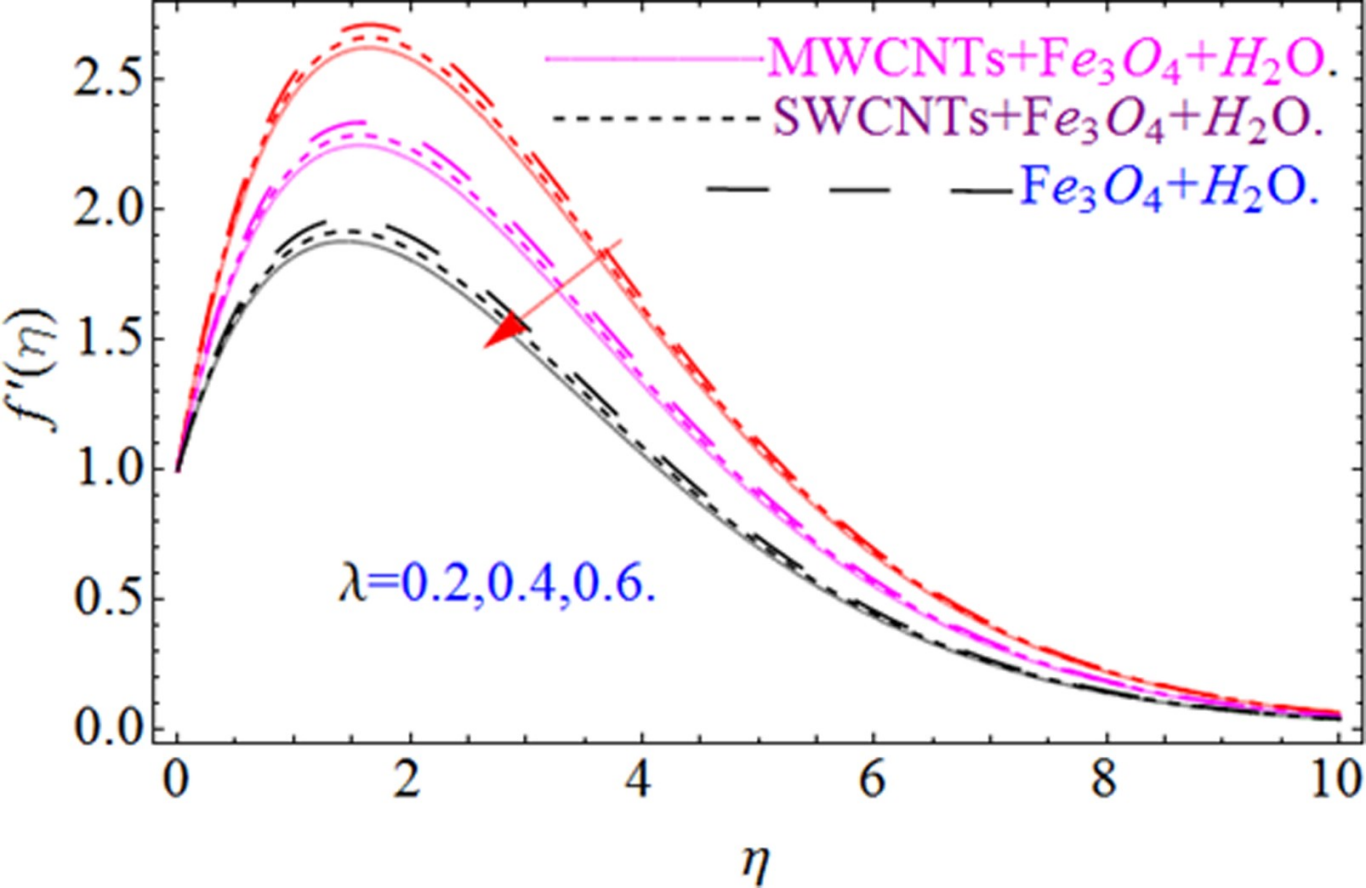


**Fig 4:**
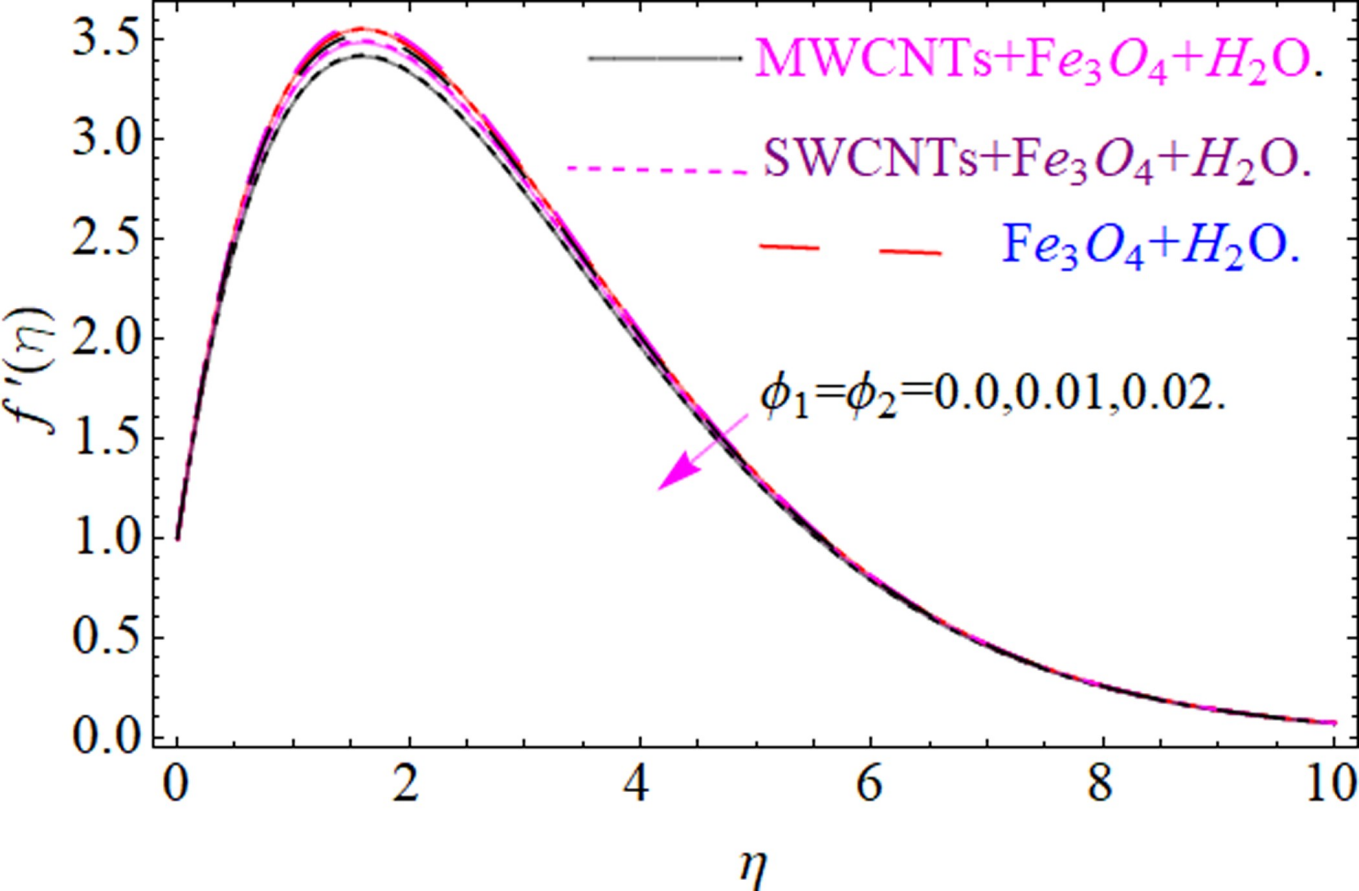


**Fig 5:**
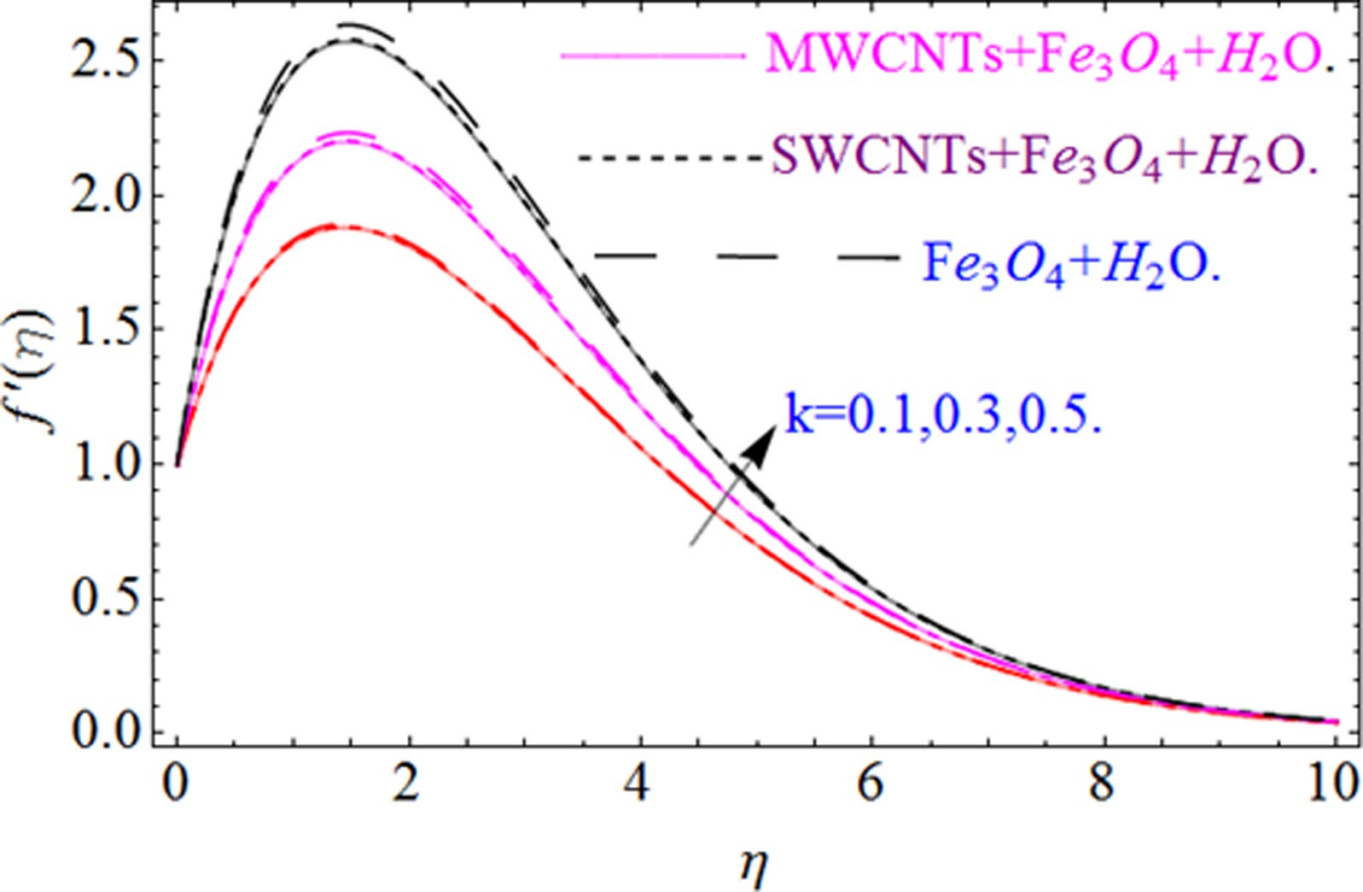


**Fig 6:**
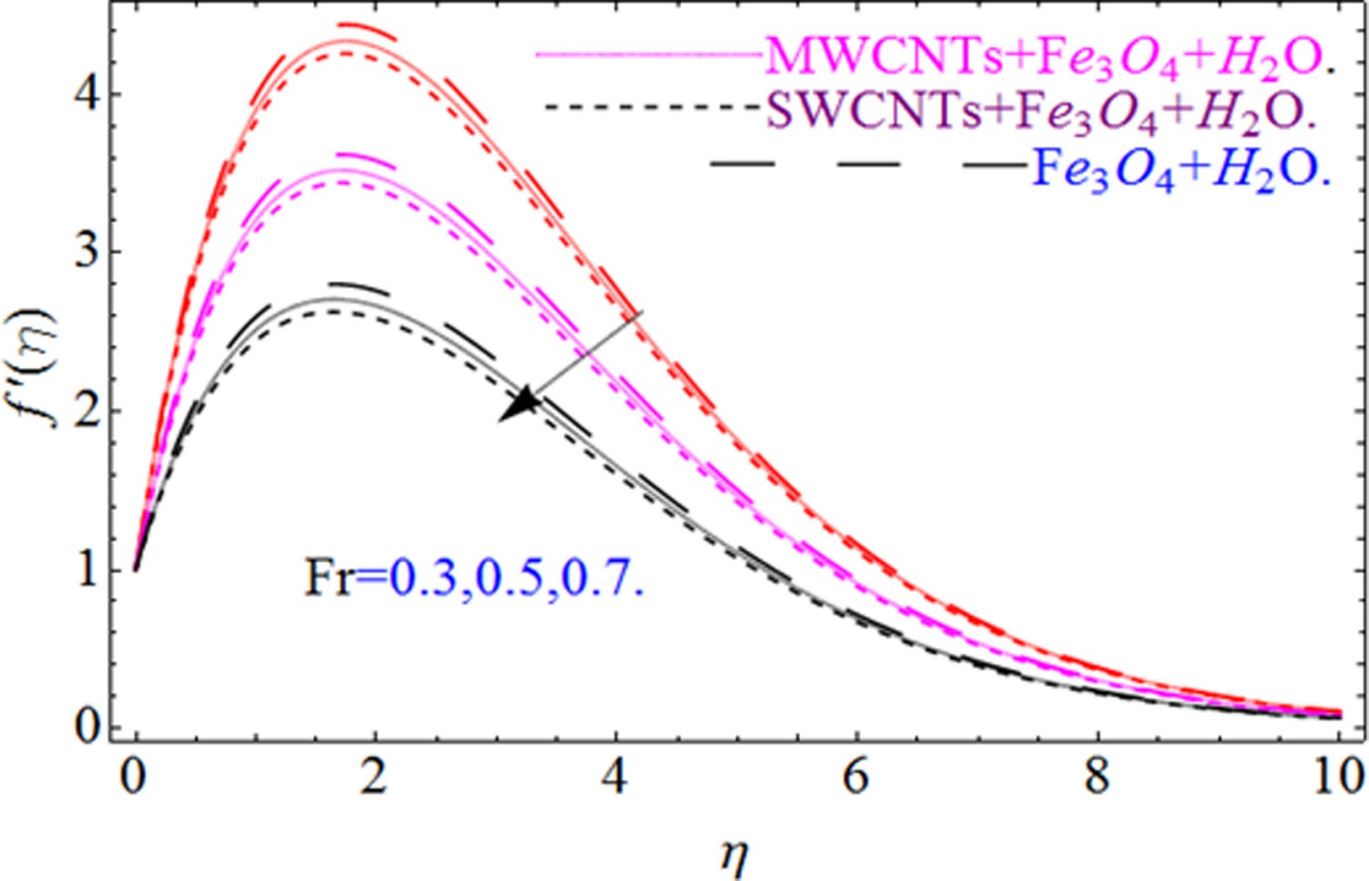


**Fig 7:**
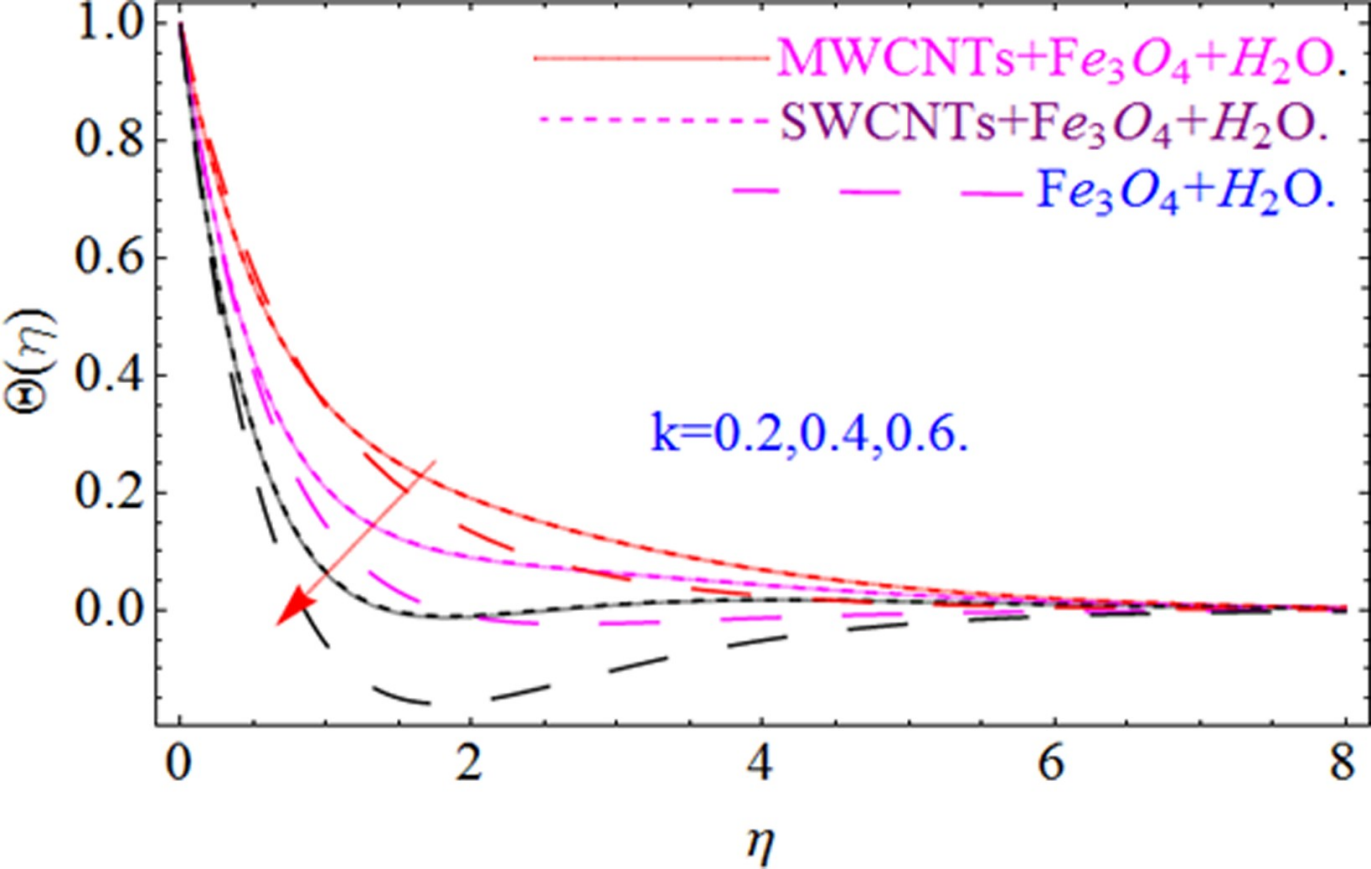


**Fig 8:**
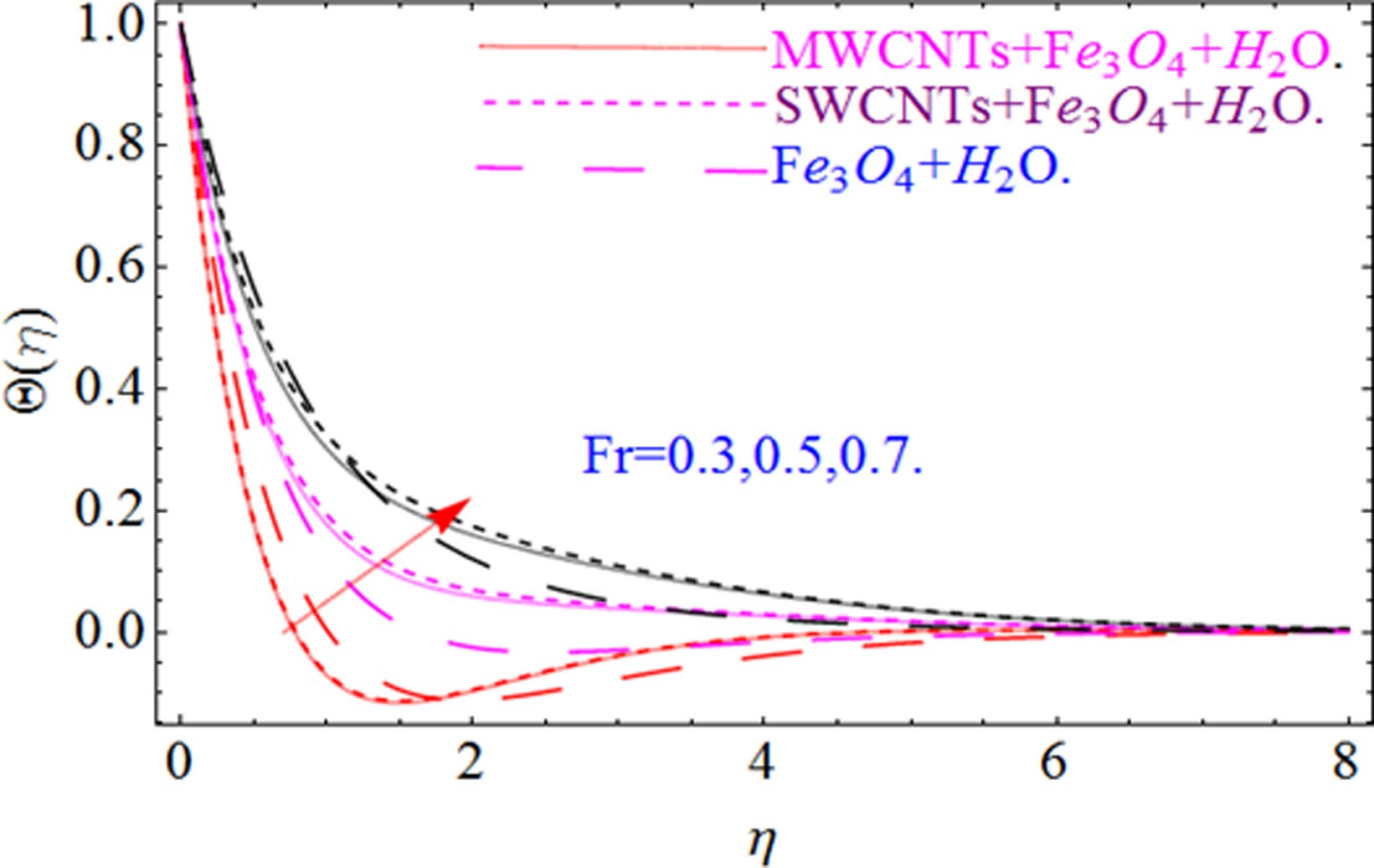


**Fig 9:**
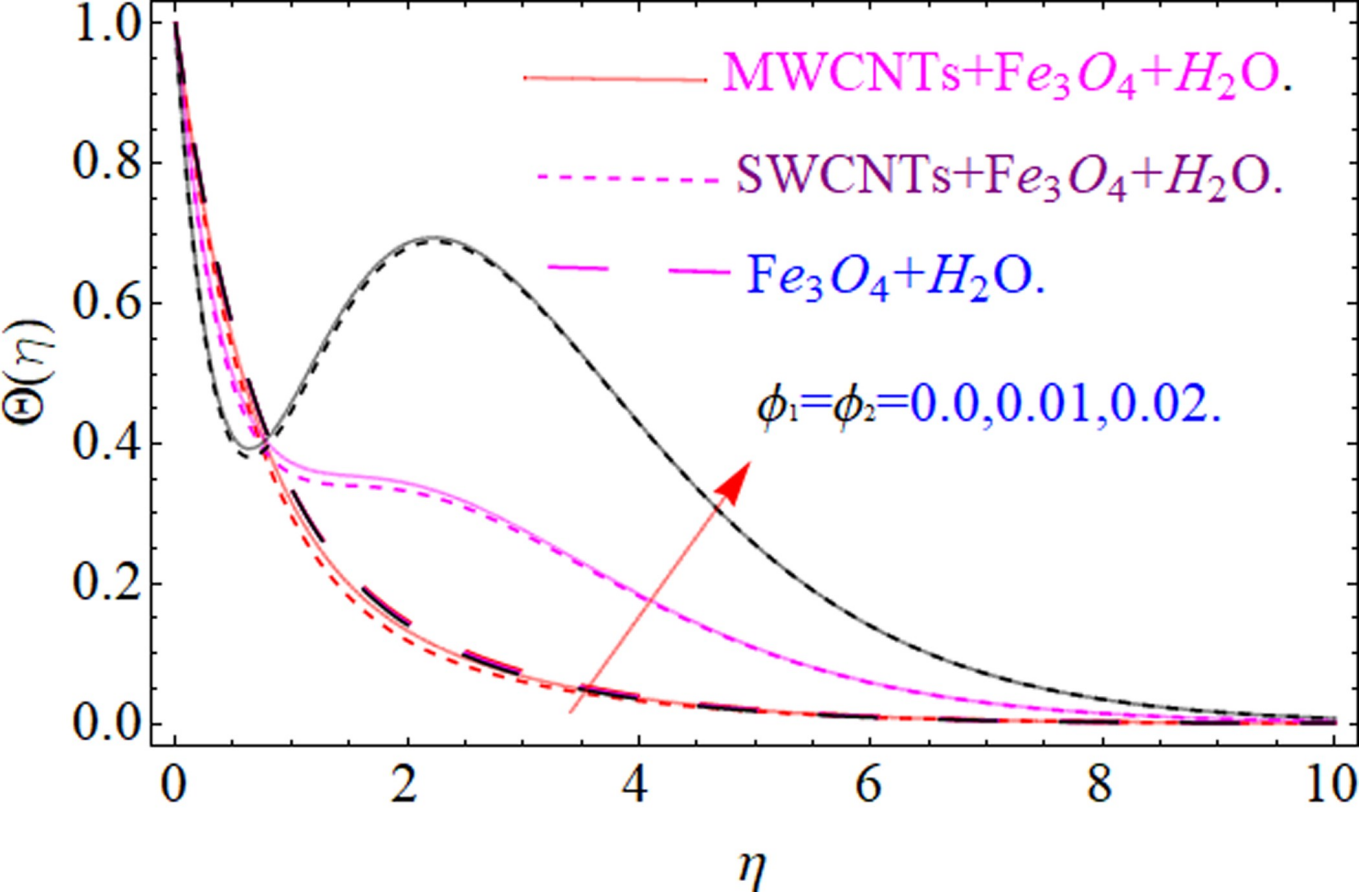


**Fig 10:**
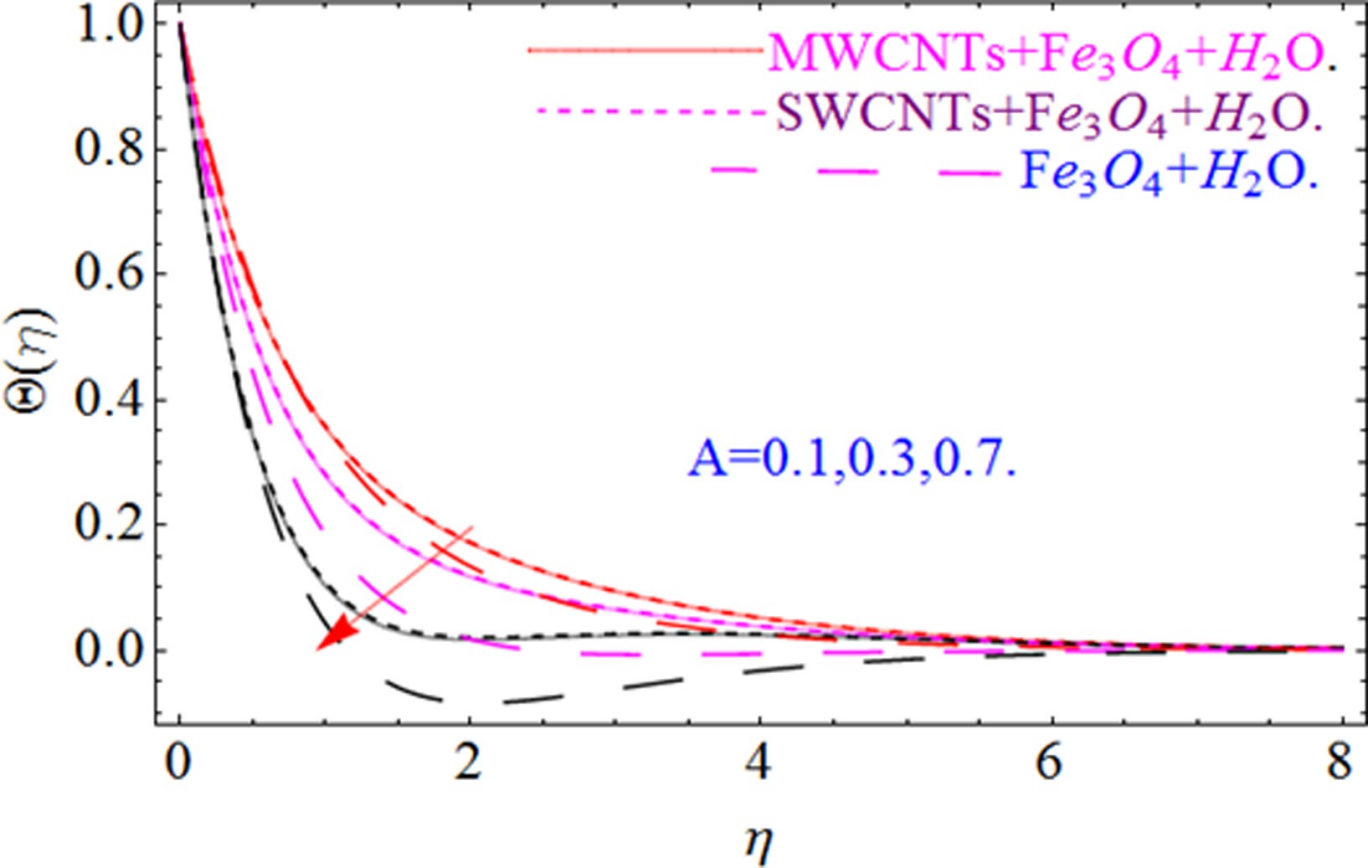


**Fig 11:**
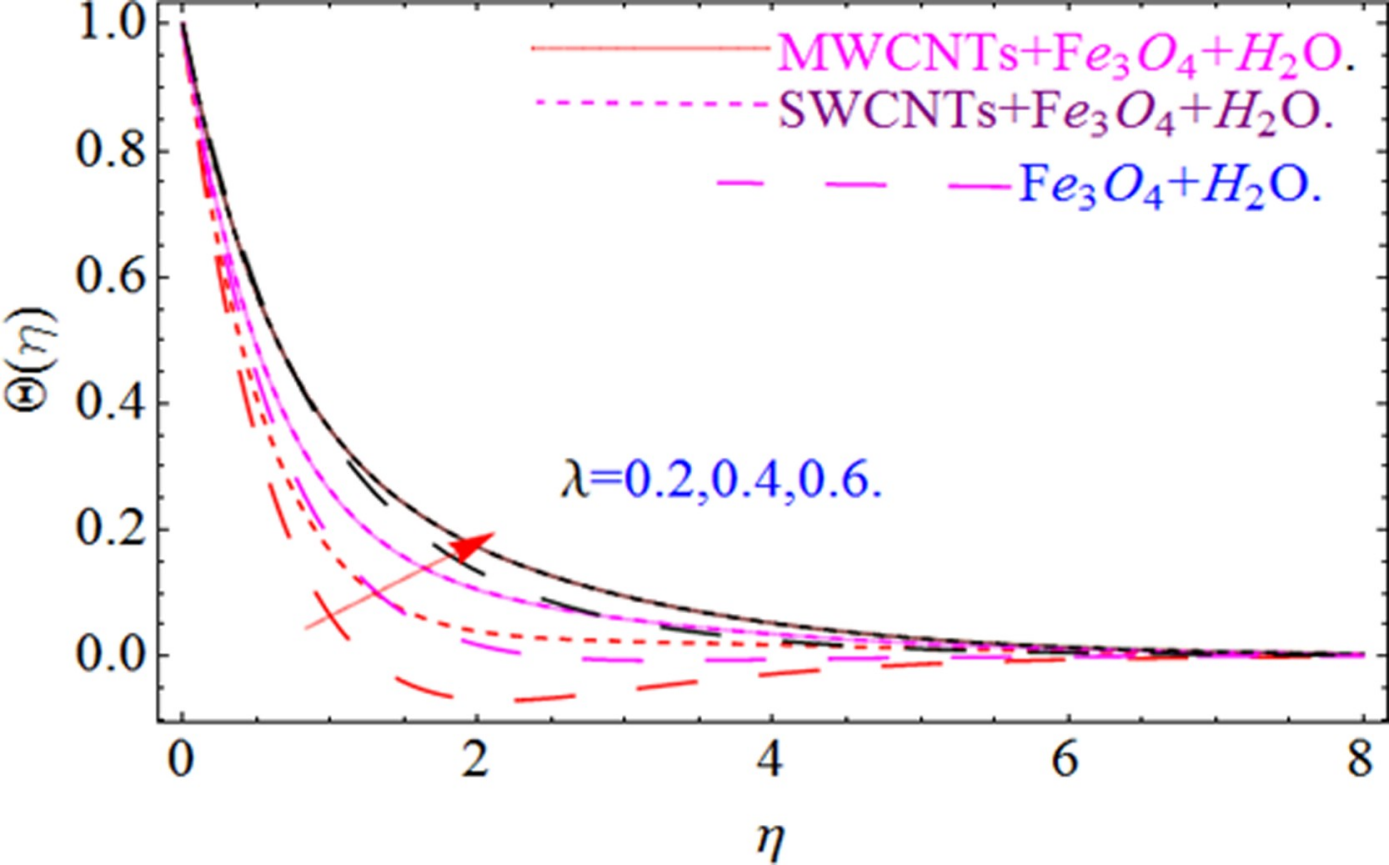


**Fig 12:**
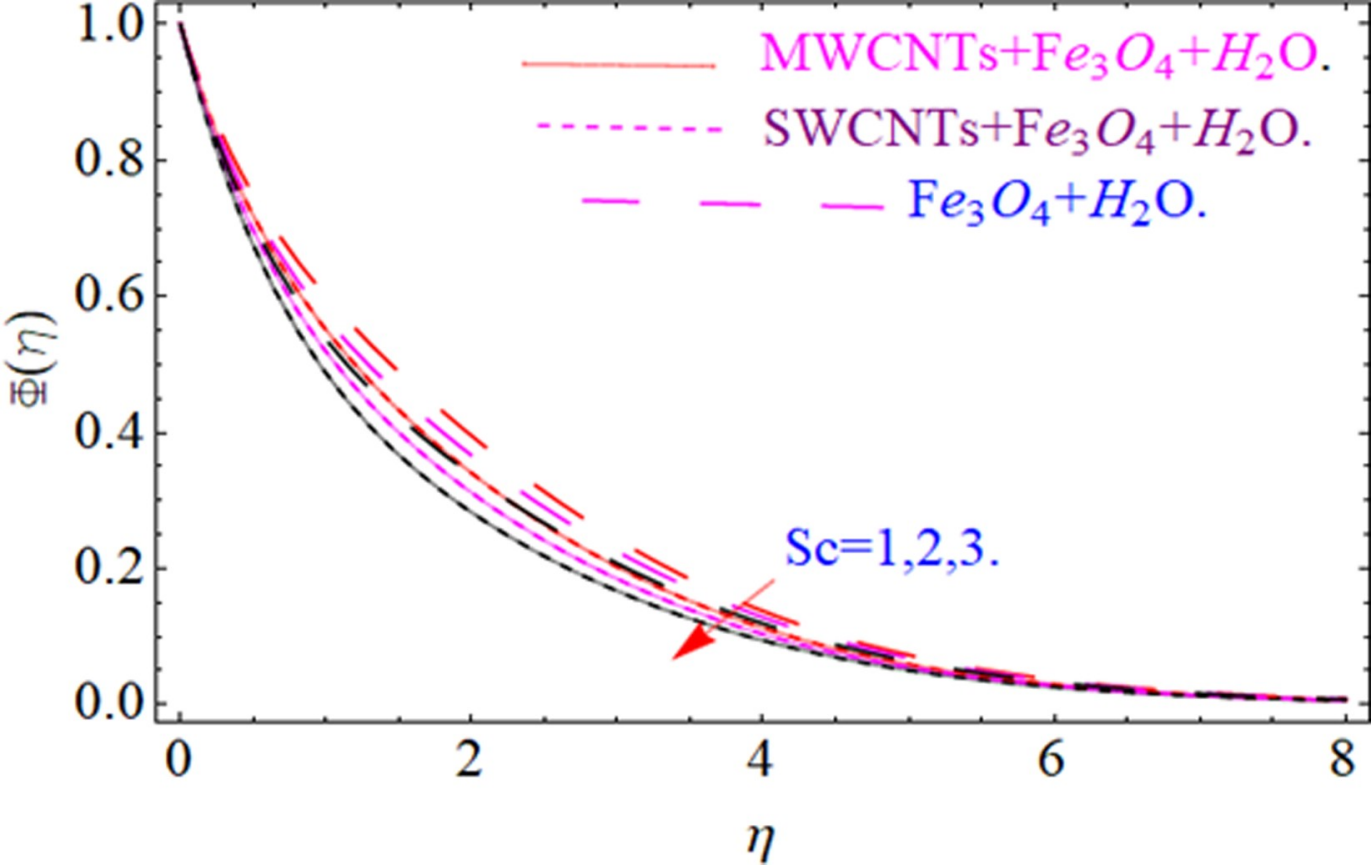


**Fig 13:**
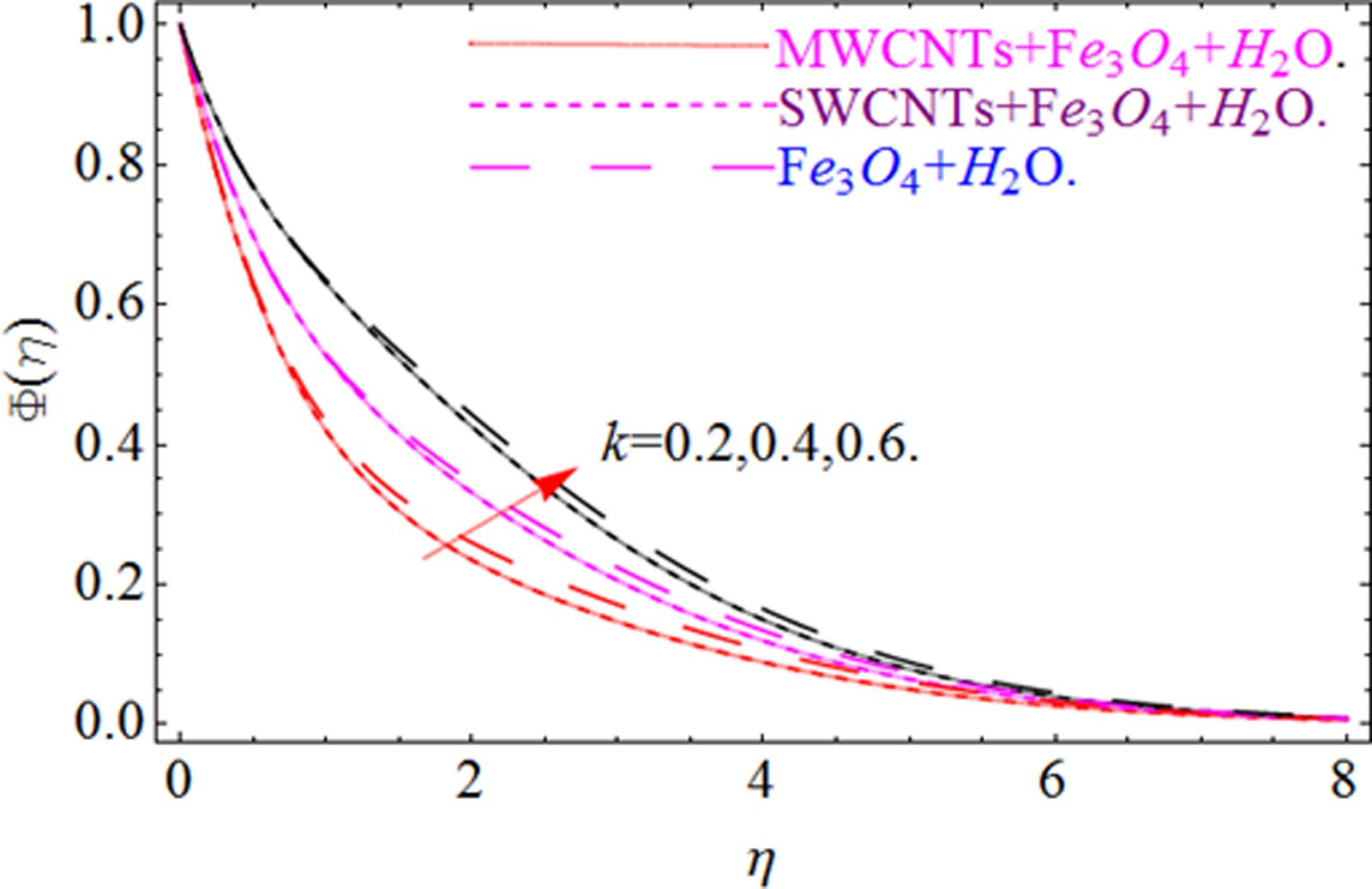


**Fig 14:**
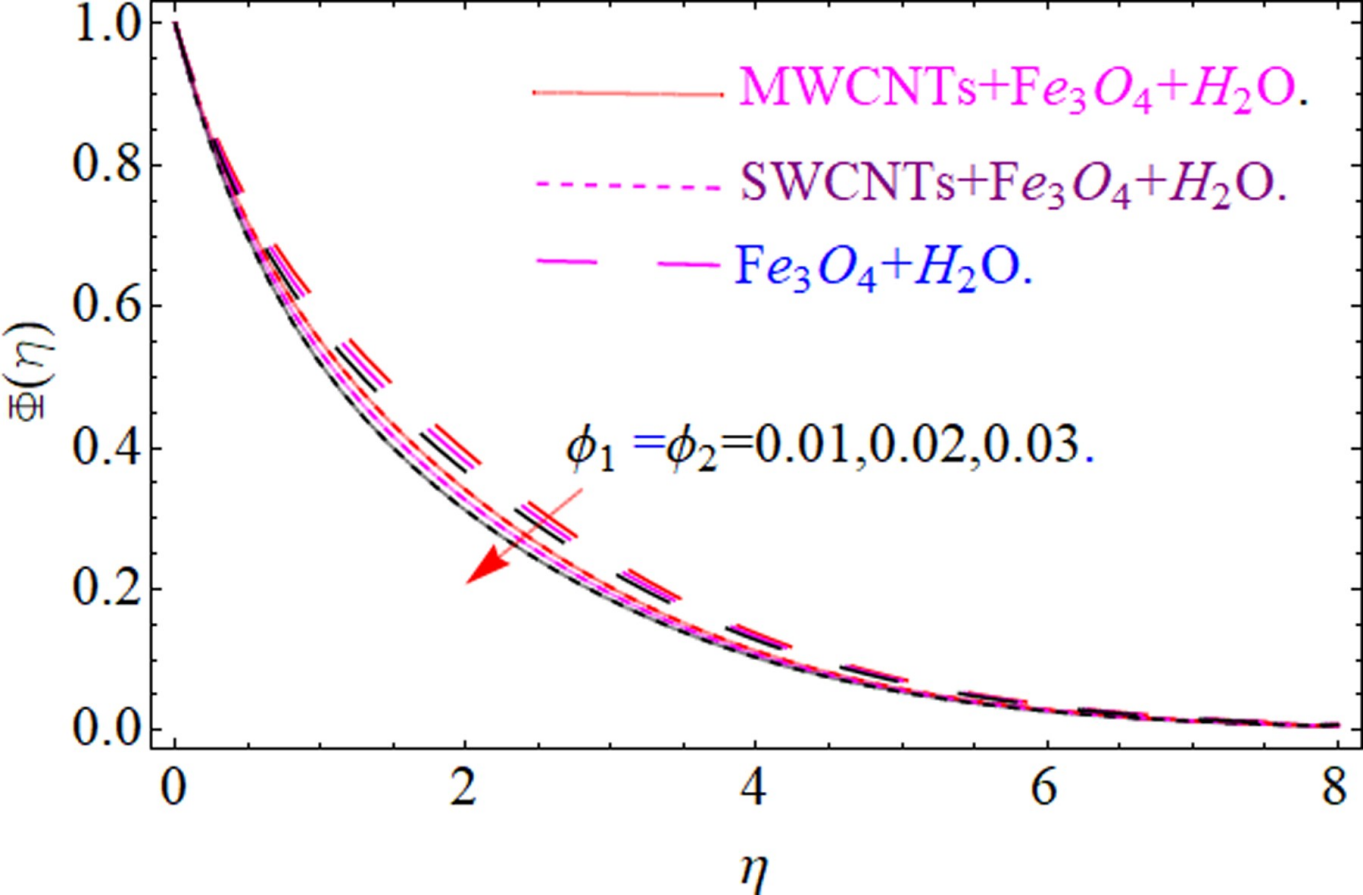


**Fig 15:**
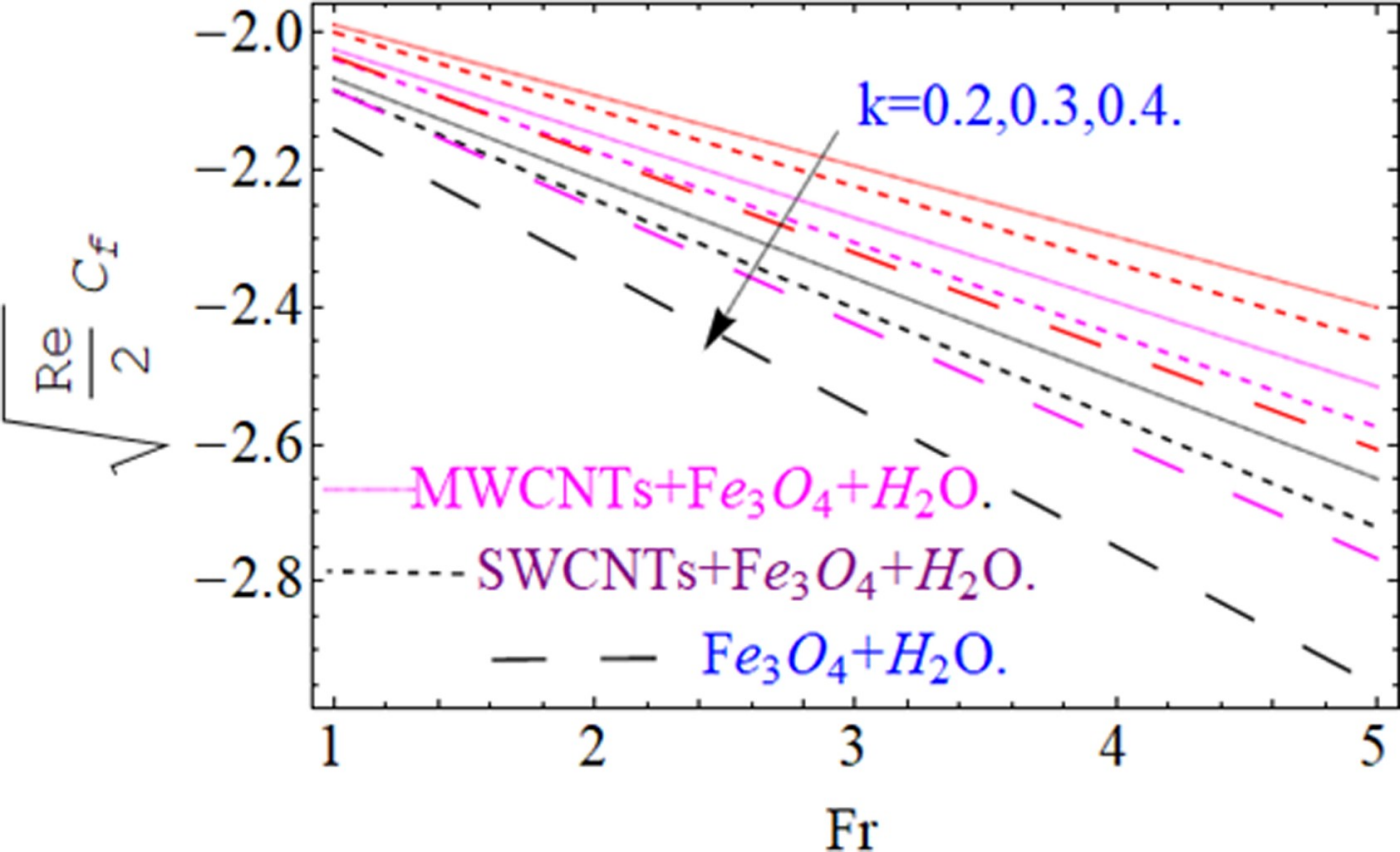


**Fig 16:**
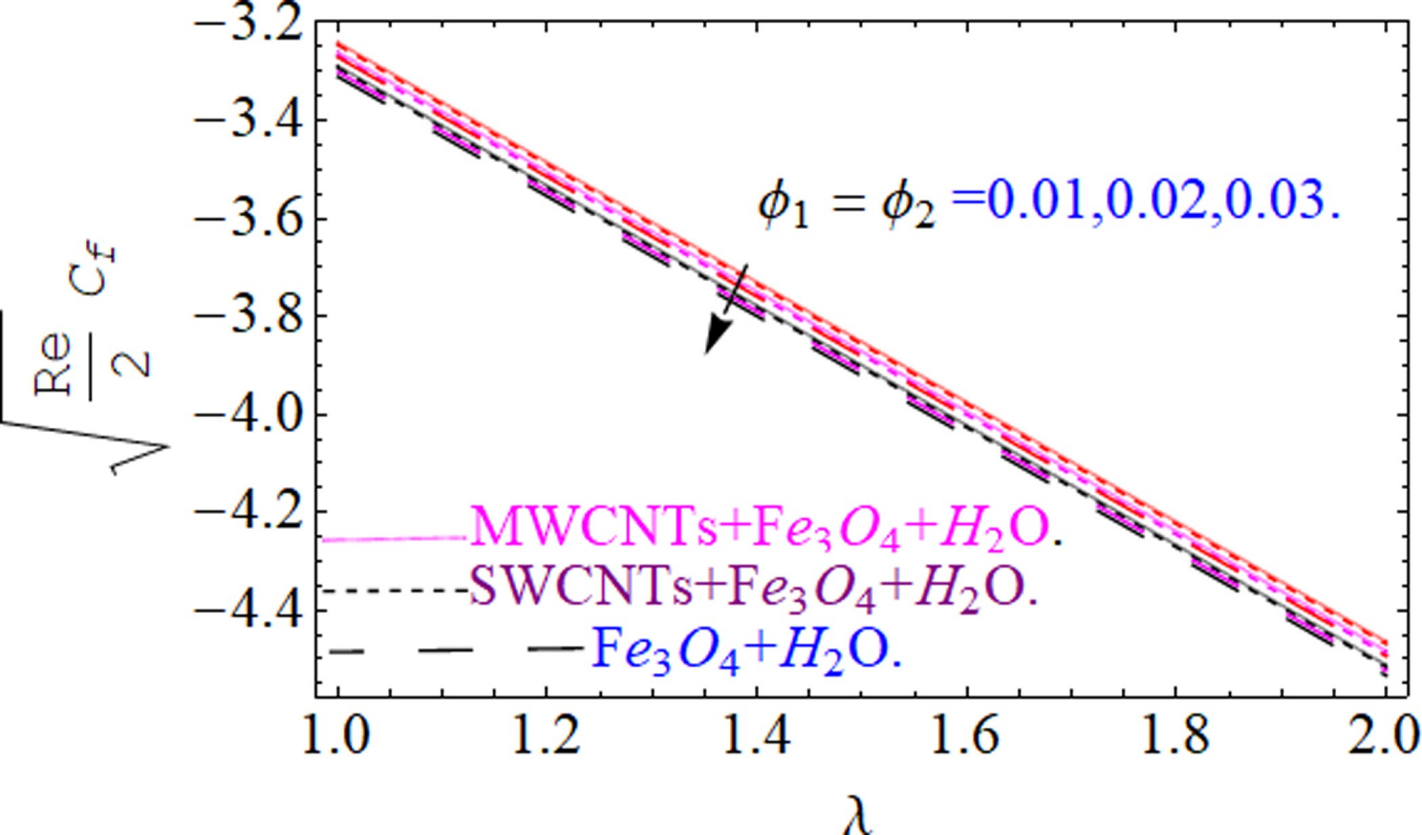


**Fig 17:**
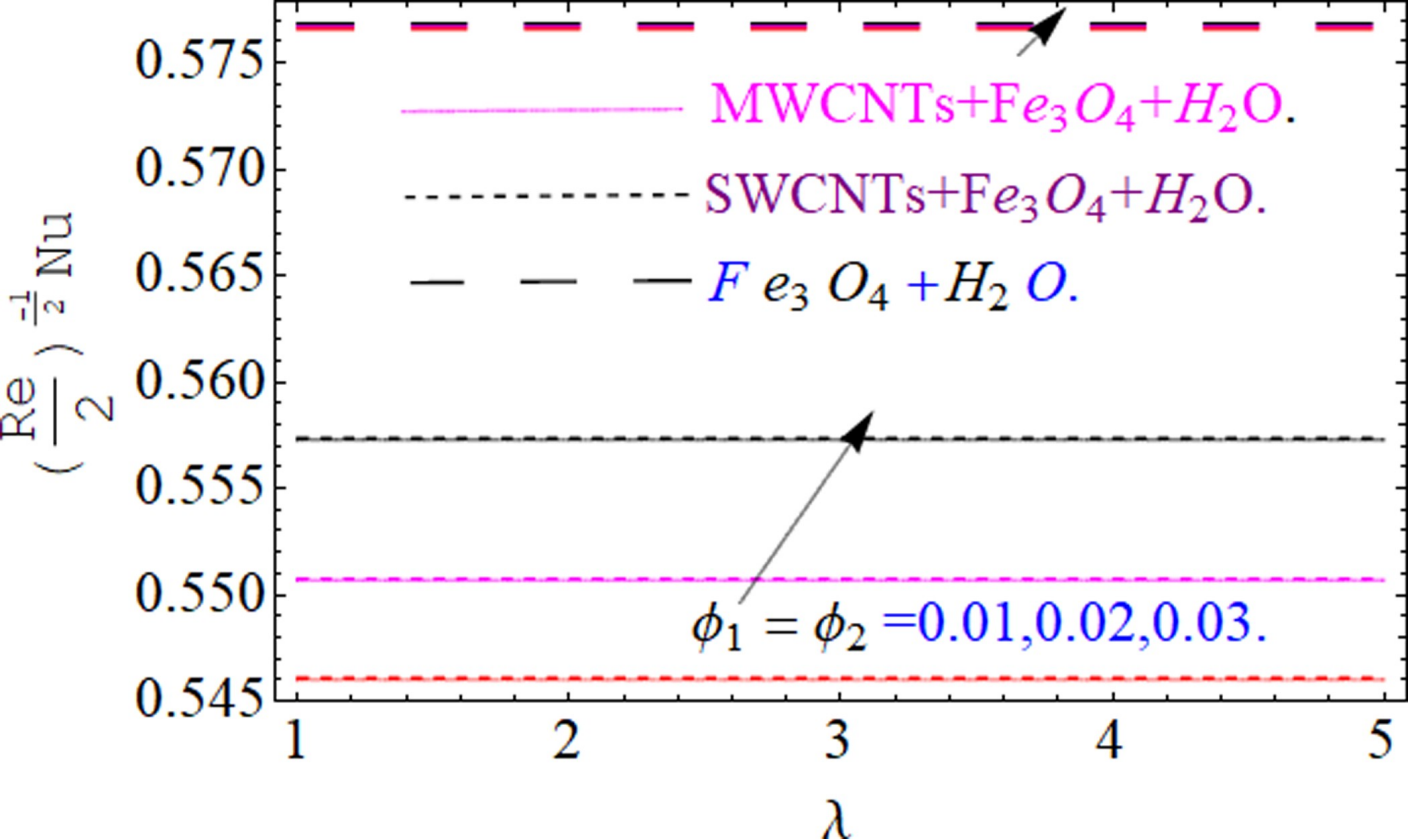


**Fig 18:**
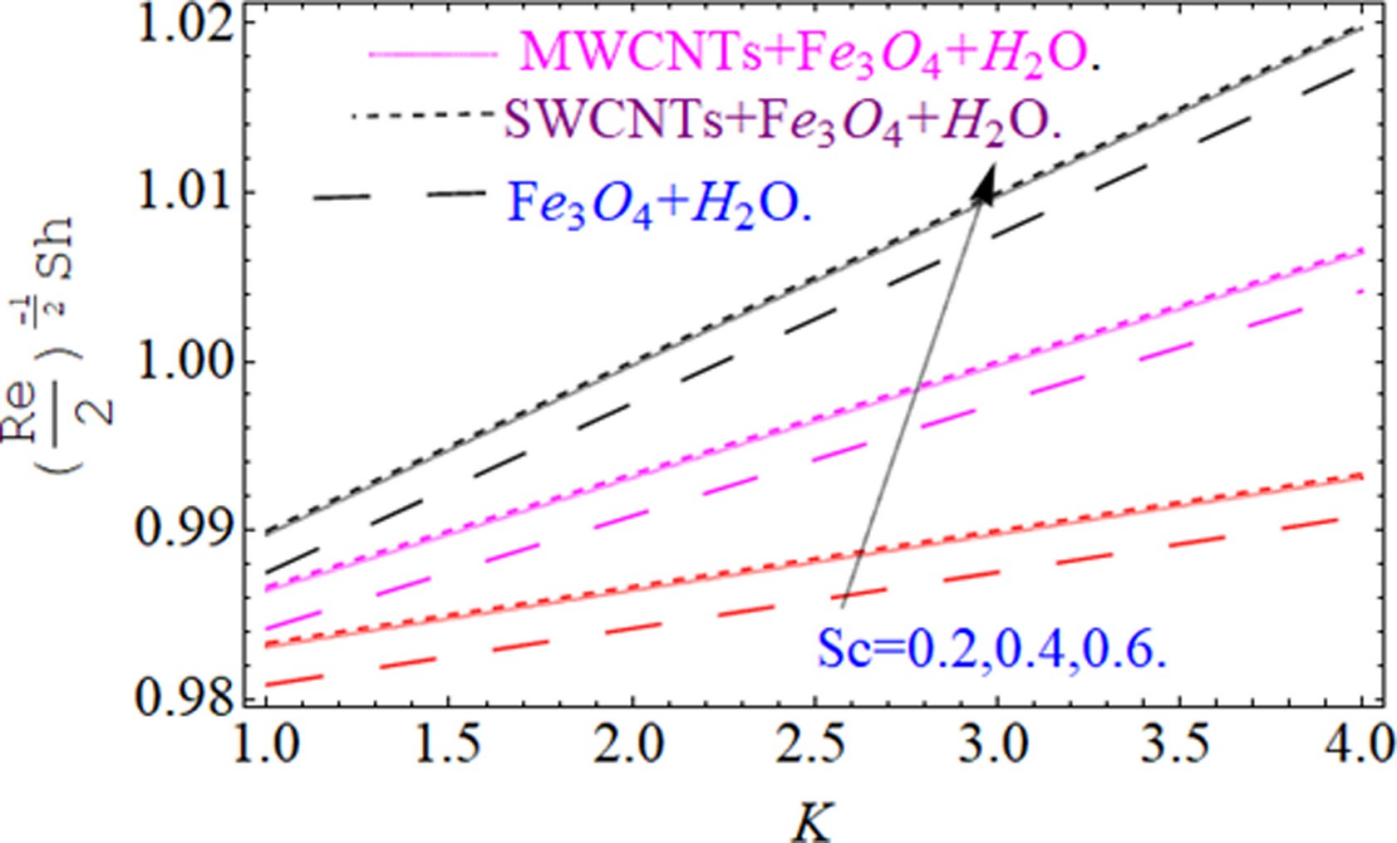


This section aims to visualize variations in temperature, flow, Nusselt number, and skin friction coefficient for involved parameters like *Fr* depicts Forchheimer number, Pr is the Prandtl number, *K* is the Curvature parameter, *λ* is the local porosity parameter and Schmidt number. Fig 1 exhibits the geometry of the flow problem. The OHAM method’s convergence has been calculated up to the 30^th^ order estimation and shown in Fig 2. Comparative investigation of nanofluid (*Fe*_3_*O*_4_+ H_2_O) and hybrid nanofluid (MWCNTs + *Fe*_3_*O*_4_ + H_2_O) and (SWCNTs + *Fe*_3_*O*_4_ + H_2_O) during studying impacts of *λ*, *Fr*, *K* and *ϕ*_1_, *ϕ*_2_ on *f*’(*η*) is visualized in Figs 3–6, respectively. Efficient trend is shown by hybrid nanomaterial (MWCNTs + *Fe*_3_*O*_4_ + H_2_O) which is followed by (SWCNTs + *Fe*_3_*O*_4_ + H_2_O) and (*Fe*_3_*O*_4_+ H_2_O). The velocity profile (*f*’(*η*)) under higher estimations of *λ* (local porosity parameter) is sketched in Fig 3. Higher estimation of *λ* (local porosity parameter) leads to decay of *f*’(*η*). Physically, *λ* is inversely proportional to darcian drag force which implies the Darcy number reduces the rise of darcian drag force, hence this is acts to enhance the permeability of the fluid flow and it leads to diminishes the fluid velocity. Enhancement of permeability rises to the resistive force within the particles so that the fluid’s velocity will be reduced. It is noted that the velocity of nanofluid (*Fe*_3_*O*_4_+ H_2_O) rapidly decreases as compared to hybrid nanofluid (MWCNTs + *Fe*_3_*O*_4_ + H_2_O) and (SWCNTs + *Fe*_3_*O*_4_ + H_2_O). Fig 4 is sketched to demonstrate the effect of *ϕ*_1_ and *ϕ*_2_ on *f*’(*η*). Higher estimation of *ϕ*_1_ and *ϕ*_2_ leads to decrement of *f*’(*η*). The transportation, cohesive forces, and excitation energy between carbon nanotubes nanoliquid and iron oxide nanoliquid decreases by increasing the quantity of volume fraction, which causes negative changes in the velocity field *f*’(*η*). The iron oxide *Fe*_3_*O*_4_ nanoliquid show greater velocity as compared to multi and single- wall carbon nanotubes, because metal atoms have free valence electrons in the valence shell than CNTs and exert a weaker force on it. Fig 5 visualizes impacts of k (curvature parameter) on velocity field *f*’(*η*)for nanofluid (*Fe*_3_*O*_4_ + H_2_O) and hybrid nanofluid (MWCNTs + *Fe*_3_*O*_4_ + H_2_O) and (SWCNTs + *Fe*_3_*O*_4_ + H_2_O). Intensification in the velocity of fluid *f*’(*η*) is examined via higher estimations of k. Physically, with the growing credit of k the radius *R* of the stretching surface increases, which improves the fluid velocity of both CNTs nanoliquid and *Fe*_3_*O*_4_ nanoliquid. Furthermore, the impacts of nanofluid (*Fe*_3_*O*_4_ + H_2_O) dominate when compared with nanomaterial hybrid nanofluid (MWCNTs + *Fe*_3_*O*_4_ + H_2_O) and (SWCNTs + *Fe*_3_*O*_4_ + H_2_O). Velocity profile *f*’(*η*) for variations in *Fr* is portrayed in Fig 6 for nanofluid (*Fe*_3_*O*_4_ + H_2_O) and hybrid nanofluid (MWCNTs + *Fe*_3_*O*_4_ + H_2_O) and (SWCNTs + *Fe*_3_*O*_4_ + H_2_O). Higher *Fr* leads to enlargement of *f*’(*η*). Physically, this mechanism happens due to the inertial effect that drags the fluid backward and accordingly with this inertial force, the speed of fluid will be decreased; therefore, the velocity profile is declined. Moreover, the impacts of hybrid nanofluid (SWCNTs + *Fe*_3_*O*_4_ + H_2_O) dominate when compared with nanomaterial hybrid nanofluid (MWCNTs + *Fe*_3_*O*_4_ + H_2_O) and nanofluid (*Fe*_3_*O*_4_ + H_2_O). Comparative investigations amongst hybrid nanomaterial (MWCNTs + *Fe*_3_*O*_4_ + H_2_O) and (SWCNTs + *Fe*_3_*O*_4_ + H_2_O) and nanomaterial (*Fe*_3_*O*_4_ + H_2_O) via impacts of *k*, *λ*, *Fr*, *A* and *ϕ*_1_, *ϕ*_2_ on temperature profile Θ(*η*) are visualized in Figs 7–11, respectively. Fig 7 depicts the k (curvature parameter) influence on the temperature Θ(*η*) profile for nanofluid (*Fe*_3_*O*_4_ + H_2_O) and hybrid nanofluid (MWCNTs + *Fe*_3_*O*_4_ + H_2_O) and (SWCNTs + *Fe*_3_*O*_4_ + H_2_O). As the curvature parameter increases, the radius of the stretching surface also increases, which ensure that the maximum number of nanoparticles will be attached to the stretching surface, which will conduct more heat, as a result, the temperature field of CNTs nanoliquid and *Fe*_3_*O*_4_ nanoliquid enhances. While opposite behavior has been observed with the increment of the Forchhemier number *Fr* in Fig 8. It is characterized that the improvement of *Fr* declines the temperature field Θ(*η*). Furthermore such reduction in temperature of nanofluid (*Fe*_3_*O*_4_ + H_2_O) is more quickly as compared to hybrid nanofluids (MWCNTs + *Fe*_3_*O*_4_ + H_2_O) and (SWCNTs + *Fe*_3_*O*_4_ + H_2_O). Fig 9 is drawn to characterize the impact of *ϕ*_1_ and *ϕ*_2_ (volume fraction of CNTs and iron oxide nanoparticles respectively) on heat transfer Θ(*η*) for hybrid nanomaterial (MWCNTs + *Fe*_3_*O*_4_ + H_2_O) and (SWCNTs + *Fe*_3_*O*_4_ + H_2_O) and nanomaterial (*Fe*_3_*O*_4_ + H_2_O). It is found that the temperature profile boost with the growing values of *ϕ*_1_ and *ϕ*_2_ for both types of nanofluids. Also the reducing trend has been observed with larger values of *A* (temperature exponent) in the temperature field Θ(*η*) captured in Fig 10. This increment in the temperature profile is more rapidly in case of nanofluid (*Fe*_3_*O*_4_ + H_2_O) as equated to hybrid nanofluids (MWCNTs + *Fe*_3_*O*_4_ + H_2_O) and (SWCNTs + *Fe*_3_*O*_4_ + H_2_O). Fig 11 visualizes the impacts of porosity parameter *λ* on temperature field Θ(*η*) for hybrid nanomaterial (MWCNTs + *Fe*_3_*O*_4_ + H_2_O) and (SWCNTs + *Fe*_3_*O*_4_ + H_2_O) and nanomaterial (*Fe*_3_*O*_4_ + H_2_O). Intensification in temperature field Θ(*η*) is examined via higher estimations of *λ*. Similarly, Comparative investigations amongst hybrid nanomaterial (MWCNTs + *Fe*_3_*O*_4_ + H_2_O) and (SWCNTs + *Fe*_3_*O*_4_ + H_2_O) and nanomaterial (*Fe*_3_*O*_4_ + H_2_O) via impacts of *k*, *λ*, *Fr*, *A* and *ϕ*_1_, *ϕ*_2_ on concentration profile Φ(*η*) are visualized in Figs 12–14, respectively. The impact of Schmidt number *Sc* and curvature parameter *k* on mass transfer Φ(*η*) are presented in Figs 12 and 13 for hybrid nanomaterial (MWCNTs + *Fe*_3_*O*_4_ + H_2_O) and (SWCNTs + *Fe*_3_*O*_4_ + H_2_O) and nanomaterial (*Fe*_3_*O*_4_ + H_2_O), respectively. The associated boundary layer thickness and concentration, increases with Schmidt number *Sc*, while opposite behavior has been examined in Fig 13. Because the mass transfer rate diminishes with increases of curvature factor *k*. Fig 14 demonstrate the influence of volume fraction indicator *ϕ*_1_ and *ϕ*_2_ for both CNTs and ferrium oxide nanoliquid on concentration profile Φ(*η*). The improvement in quantity of iron oxide nanoparticles and carbon nanotubes actually slows down the mass transfer rate, because the fluid average viscosity becomes dense. Thus the concentration profile decline with growing credit of *ϕ*_1_ and *ϕ*_2_.The surface drag force Re2Cfx for carbon nanoliquid and *Fe*_3_*O*_4_ are declared via Figs 15 and 16. It is perceived that the skin friction reduces with the positive increment of curvature factor *k* and volume friction parameter *ϕ*_2_. Fig 17 illustrate the numerical outcomes for Nusselt number LS(Re2)−12Nux. It is examined that the heat transmission rate improved with the improvement of the carbon nanotubes *ϕ*_2_ quantity in base fluid. The Sherwood number LS(Re2)−12Shx is the improving function of the Schmidt number shown in Fig 18.

The thermo physical properties of the solid materials and base fluids are shown in the Table 1. The OHAM method’s convergence has been calculated up to the 30^th^ iteration and displayed in Table 2. The comparison of the present work with the available literature is revealed in Table 3. In Table 4, the enhancement in heat transfer rate for the SWCNTs, MWCNTs and Iron Oxide water based nanofluids have been calculated in %. According to the obtained results, iron oxide nanoparticles are more efficient in enhancing the heat transfer rate. Similarly, the SWCNTs are comparatively more reliable for the heat transfer rate as compared to MWCNTs.

## 5. Conclusion

In the existing study, we talked about the Darcy Forchheimer flow of hybrid nanoliquid due to an extending curved surface. The flow model is arranged in the form of differential equations containing momentum and energy equation. The analytic arrangement of modeled equations is further set up by the “Homotopy analysis method” (HAM). The concluded findings are pointed out as:

- The temperature and velocity fields, both show similar behaviors for rising values of volume fraction parameters of CNTs and *Fe*_3_*O*_4_.
- To keep maintain the coolant level in the industrial equipment’s uses of *CNT* and *Fe*_3_*O*_4_ nanoparticles is significantly useful.
- The reducing trend has been observed in temperature with larger values of A (temperature exponent).
- The improving credit of k (curvature parameter) results in high fluid velocity, while reducing the temperature of the fluid.
- But Forchheimer number shows the opposite trend for temperature and velocity profile, because with increases of Fr the fluid velocity reduces, while the temperature profile enhances.
- The % increase in the heat transfer rate observed using the hybrid nanofluid. It is perceived that the hybrid nanofluid enhance the heat transfer rate (3.1461%) as compared to the other traditional fluids.
- The conclusion states that the hybrid nanofluids are the fast agents for the heat transfer analysis as compared to the common fluids.

**Table:** 

**Nomenclature**	
*u*, *v*	Velocity component
*r*, *s*	Space coordinates
*A*	Temperature exponent
*k*_*f*_	Thermal conductivity of base fluid
*a*_*f*_	Base fluid thermal diffusivity
*μ*_*f*_	Dynamic viscosity
*v*_*f*_	Kinematic viscosity
*F*	Nonporous inertia parameter
*H*	Dimensionless pressure
*λ*	Local porosity parameter
*T*_*w*_	Wall temperature
*Fr*	Forchheimer numbers
*K*	Curvature parameter
Θ	Dimensionless temperature
*ϕ*	Volume friction
*C*_*f*_	Skin friction Coefficient
*C*_*b*_	Drag coefficient
*v*_*hnf*_	Nanofluids kinematic viscosity
*μ*_*hnf*_	Dynamic viscosity of nanofluids
*ρ*_*hnf*_	Nanofluids density
*Nu*_*s*_	Local Nusselt number
Re_*s*_	Local Reynold number
K*	Porous media

## Supporting information

S1 Data (ZIP)
